# Multivesicular liposomes for sustained release of bevacizumab in treating laser-induced choroidal neovascularization

**DOI:** 10.1080/10717544.2018.1474967

**Published:** 2018-06-05

**Authors:** Hongjie Mu, Yiyun Wang, Yongchao Chu, Ying Jiang, Hongchen Hua, Liuxiang Chu, Kaili Wang, Aiping Wang, Wanhui Liu, Youxin Li, Fenghua Fu, Kaoxiang Sun

**Affiliations:** a School of Pharmacy, Collaborative Innovation Center of Advanced Drug Delivery System and Biotech Drugs in Universities of Shandong, Key Laboratory of Molecular Pharmacology and Drug Evaluation, Ministry of Education, Yantai University, Yantai, Shandong Province, People’s Republic of China;; b State Key Laboratory of Long-Acting and Targeting Drug Delivery System, Shandong Luye Pharmaceutical Co., Ltd., Yantai, Shandong Province, People’s Republic of China

**Keywords:** Bevacizumab, choroidal neovascularization, multivesicular liposomes, bioactivity, intravitreal injection, vitreous humor

## Abstract

Bevacizumab is an anti-vascular endothelial growth factor drug that can be used to treat choroidal neovascularization (CNV). Bevacizumab-loaded multivesicular liposomes (Bev-MVLs) have been designed and developed to increase the intravitreal retention time of bevacizumab and reduce the number of injection times. In this study, Bev-MVLs with high encapsulation efficiency were prepared by double emulsification technique, and antibody activity was determined. The results revealed that 10% of human serum albumin (HSA) could preserve the activity of bevacizumab. *In vitro* release of Bev-MVLs appeared to be in a more sustained manner, the underlying mechanisms of Bev-MVLs indicated that bevacizumab was released from MVLs through diffusion and erosion. Results of sodium dodecyl sulfate-polyacrylamide gel electrophoresis (SDS-PAGE) demonstrated that bevacizumab could retain its structural integrity after being released from MVLs *in vitro*. *In vivo* imaging was used to evaluate the retention time of antibody in rat eyes, while pharmacokinetic analysis was performed on rabbit eyes. These results indicated that Bev-MVLs exhibited sustained release effects as compared to bevacizumab solution (Bev-S). Bev-MVLs could effectively inhibit the thickness of CNV lesion as compared to Bev-S at 28 days after treatment. Furthermore, these data suggest that Bev-MVLs are biologically feasible to increase the retention time of bevacizumab in vitreous humor. This novel Bev-MVLs may therefore serve as a promising sustained release drug delivery system for the treatment of CNV.

## Introduction

Choroidal neovascularization (CNV) is characterized by the abnormal growth of new blood vessel extending from the choroid into the subretinal or retinal pigment epithelium (RPE) space through a break in Bruch’s membrane (Campochiaro et al., 1999). It is a proliferative ocular disease and is accompanied by various complications, including age-related macular degeneration (AMD), the leading cause of vision loss among the elderly in Western countries (VanNewkirk et al., 2000). Vascular endothelial growth factor (VEGF) has been found to play an important role in neovascular growth (Carmeliet et al., 1996). Overexpression of VEGF may leak fluid and blood, cause apoptotic cell death, and ultimately lead to the formation of CNV (Ardeljan & Chan, 2013). In fact, anti-VEGF drugs have shown great promise for the treatment of CNV (Angkawinitwong et al., 2017; Neves Cardoso et al., 2017). These medications are widely applied in clinical practice, with reasonable efficacy and safety (Mi et al., 2011; Kodjikian et al., 2013).

Bevacizumab (Avastin^®^) is a full-length recombinant humanized monoclonal antibody, which can bind all of the VEGF isoforms. Bevacizumab can be used to treat colon cancer, through the inhibition of tumor neovascularization. Although intravitreal injection with bevacizumab has been widely accepted for the treatment of AMD (Spaide et al., 2006), this drug is of off-label use and its half-life in vitreous and aqueous humor is 3–5 days (Bakri et al., 2007). Therefore, a higher dosage or more frequent administration is needed to reach the minimum effective concentration, which consequently increases the risk of toxicity in AMD patients (Li et al., 2013; Kaur & Kakkar, 2014). Indeed, the health care costs of cataract formation, vitreous hemorrhage, endophthalmitis, and other complications should be considered when developing a sustained release drug delivery system.

More recently, biodegradable polymeric material is considered to be the most promising matter of choice for its sustained release capability. Several drug delivery systems have been developed to treat intraocular diseases and prolong drug release in vitreous humor, based on implants (Haghjou et al., 2011), microspheres (Mordenti et al., 1999), hydrogel (Xu et al., 2013), and nanoparticles (Li et al., 2012; Varshochian et al., 2013). However, the burst release of drugs in sustained delivery systems is to be solved. Moreover, the acidic condition due to polymer degradation may inactivate and degenerate macromolecules (Jiskoot et al., 2012; Hasan et al., 2015), leading to intraocular tissue inflammation and even blindness if left untreated.

Multivesicular liposomes (MVLs) are a specific depot delivery system, which consist of numerous discontinuous internal aqueous compartments separated by several nonconcentric lipid bilayers (Kim et al., 1983). The volume ratio of 95:5 (aqueous:lipid) of MVLs is rendered, indicating the high encapsulation efficiency for water-soluble drugs. In addition to its unique structure, the typical size of MVLs particles is in the range of 1–100 μm. This is particularly important for systemic delivery applications to avoid the rapid clearance by tissue macrophages. MVLs can also form a drug-depot to prolong the release time at the site of injection and reduce the initial burst release of drugs. Furthermore, nonconcentric lipid layers can improve the stability of protein or peptide (Kohn et al., 1998; Mantripragada, 2002). Meanwhile, the degradation products of MVLs are nontoxic, nonirritating, biocompatible, and biodegradable. Therefore, it is suggested that MVLs encapsulated bevacizumab may have potential applications in the treatment of CNV.

In this study, bevacizumab-loaded multivesicular liposomes (Bev-MVLs) were prepared by double emulsion method to solve the initial burst release of antibody and prolong the release time of bevacizumab. Moreover, stabilizer was incorporated to preserve the biological activity of bevacizumab. Both *in vitro* and *in vivo* studies were carried out to investigate the beneficial therapeutic effects of MVLs.

## Experimental section

### Materials

Bevacizumab (Avastin^®^) was purchased from Genentech/Roche (San Francisco, CA, USA). Polyvinyl alcohol (PVA 22000), human serum albumin (HSA), and bovine serum albumin (BSA) were purchased from Sigma-Aldrich (St. Louis, MO, USA). Egg yolk phospholipids (EPC), soybean phospholipids (SPC), 1,2-dioleoyl-sn-glycero-3-phosphocholine (DOPC), and 1,2-dipalmitoyl-sn-glycero-3-phosphoglycerol (DPPG) were purchased from Lipoid GmbH (Ludwigshafen, Germany). Triolein, cholesterol, L-lysine, sucrose, glucose, and other materials were purchased from Sinopharm Chemical Reagent Co., Ltd. (Shanghai, China). VEGF_165_ and goat anti-human IgG (Fc-specific) secondary antibody (horseradish peroxidase [HRP]) were purchased from Beijing Sino Biological Inc. (Beijing, China).

### Preliminary studies

#### Enzyme-linked immunosorbent assay (ELISA)

Sandwich ELISA was used to determine the concentrations of bevacizumab. VEGF_165_ was immobilized on a 96-well assay plate at 1 μg/mL in 0.15 M phosphate-buffered saline (PBS, pH 7.4). The plate was incubated overnight at 4 °C and subsequently blocked with 1% BSA in PBS-Tween (pH 7.4) at 37 °C for 1 h. Serial dilutions of samples were prepared and added on the plates incubated for 1 h. The goat anti-human IgG (Fc-specific) secondary antibody conjugated to HRP was used to detect bevacizumab. Between each step, the plates were rinsed five times with PBS (pH 7.4). TMB substrate was used to allow the color development and the reaction was stopped by adding 1 M H_3_PO_4_. The optical density of each well was measured at 450 nm by a plate reader (M2e, Molecular Devices, Sunnyvale, CA, USA).

#### Effect of additives on antibody activity

In our preliminary study, we found that the bevacizumab activity was hampered by the presence of organic/aqueous interface during preparation step (Lee et al., 2002). In order to investigate the effect of additives on antibody activity, each additive was added with bevacizumab during the emulsification process in the absence of lipid phase. Initially, 0.5 mL of bevacizumab solution (1% w/v) was mixed with various amounts of additive (in pH 7.4 PBS, containing 5% w/v sucrose), by dropping into 0.5 mL organic phase (chloroform:diethyl ether 1:1, v/v) and stirring for 1 min at 500 rpm in ice bath. The first emulsion was incorporated into 2 mL of the second aqueous phase (containing 4% w/v glucose and 40 mM lysine) and stirred for 3 min at 500 rpm in ice bath. Then, the multiple emulsion was centrifuged at 4500 rpm for 15 min, and the separated aqueous phase was collected. Bev-S with no additive was used as reference group. The biological activity of bevacizumab was determined by ELISA.

### Preparation of FITC-labeled bevacizumab (FB)

A total of 1 mL of Bev-S (25 mg/mL) was dialyzed three times in carbonate buffer solution (pH 9.0) at 4 °C. Then, 1 mg of FITC was dissolved separately in 1 mL DMSO. The mixed FITC solution was dropped into Bev-S and followed by incubation at 4 °C for 8 h. Subsequently, the resulted FB mixture was dialyzed against PBS (pH 7.4) to remove free FITC molecules.

### Preparation of MVLs

Bev-MVLs were prepared by double emulsification method (w/o/w) as described previously (Katre et al., 1998). Briefly, 2 mL of 1% w/v bevacizumab (containing 5% w/v sucrose and 10% w/v HSA) was added into a lipid solution (chloroform:diethyl ether 1:1, v/v) containing 10 mg/mL DOPC, 2 mg/mL DPPG, 8 mg/mL cholesterol, and 2 mg/mL triolein, with a volume ratio of 1:1 (aqueous solution:lipid solution). The mixture was then homogenized in ice bath for 6 min at 10,000 rpm. The resulted water-in-oil emulsion was added dropwise into the second aqueous solution (2-fold increase in the volume of first emulsion) containing 4% w/v glucose and 40 mM lysine. In order to get the w/o/w emulsion, the mixture was homogenized at 6500 rpm for 1 min. Finally, chloroform and diethyl ether were removed by rotary evaporation at 37 °C. Likewise, MVLs encapsulated FB and consisted of other natural phospholipids were prepared according to the same procedure described earlier.

### Encapsulation efficiency and antibody activity of Bev-MVLs

High performance liquid chromatography (HPLC) was used to determine the concentration of bevacizumab. Affinity chromatography column (Protein A column [staphylococcus protein A, SPA]) combined with the immune globulin (mainly IgG) was applied to the quantitative analysis of bevacizumab. To determine the amount of encapsulation (D_1_), 2 mL Bev-MVLs suspension was centrifuged at 3000 rpm for 10 min, the precipitant was washed by normal saline (NS), centrifuged for three times, and followed by dissolution in 10% triton X-100. Another 2 mL suspension was dissolved in 10% triton X-100 to obtain the total amount (D_2_). The encapsulation efficiency of Bev-MVLs was calculated by D_1_:D_2_. Bev-MVLs membranes were then ruptured by 10% triton X-100 in order to determine the bevacizumab activity in Bev-MVLs. The concentration of bevacizumab was then measured by HPLC, and the same concentration of Bev-S was used as a reference.

### Morphology and size distribution of Bev-MVLs

FB-MVLs and Bev-MVLs were examined by an inverted fluorescence microscope at 400× magnification, under both bright-field and fluorescence observations (AxioVert A1, Carl Zeiss, Gottingen, Germany). The particle size distribution of Bev-MVLs was measured using a laser particle analyzer (Mastersizer 3000E, Malvern Instruments Ltd., Malvern, UK). Bev-MVLs were then frozen and lyophilized overnight. The morphology of Bev-MVLs was observed by scanning electron microscopy (S-4800, Hitachi, Tokyo, Japan).

### *In vitro* drug release assay

The effect of different factors on drug release behavior was investigated. The precipitant washed by NS was transferred into a 5 mL tube filled with release medium, and then incubated at 37 °C under general dynamic conditions. At scheduled time intervals, the supernatant of release medium was withdrawn after centrifugation, and equivalent amount of fresh medium was replaced. The concentration of drug released at each time interval was analyzed by HPLC as aforementioned. The accumulative percentage of Bev-MVLs released was calculated basing on the ratio of drug released and total amount of drug. Subsequently, the morphological change of MVLs particles was observed during the *in vitro* release at various time points.

### Structural stability of bevacizumab released from MVLs

Sodium dodecyl sulfate-polyacrylamide gel electrophoresis (SDS-PAGE) was used to study the structural stability of bevacizumab released from MVLs in non-reducing conditions at scheduled intervals. All the samples were electrophoresed on 10% separating gel and 4% stacking gel. After electrophoretic separation, Coomassie Brilliant Blue staining was used to detect and visualize proteins.

### *In vivo* imaging in rats

Cy7-NHS, a hydrophilic fluorescence dye ester, was labeled on bevacizumab. The Cy7-labled bevacizumab (CB) was then encapsulated in MVLs. Six SD rats were divided into two groups: CB and CB-MVLs groups. All the rats were anesthetized by an intraperitoneal injection of 10% chloral hydrate solution (0.3 mL/100 g). Meanwhile, tropicamide eyedrop was used to dilate the rat pupils. Subsequently, the left eyes were intravitreally injected with 0.25 mg/10 μL CB and CB-MVLs, respectively. The rats were monitored on 0.5, 1, 3, 7, and 14 days, by using *In-Vivo* Imaging System (FX Pro, Carestream Health, Rochester, NY, USA). Both X-ray and fluorescence images (excitation 720 nm, emission 790 nm) were obtained and overlaid. Finally, the position and intensity of each fluorochrome were determined over time.

### Pharmacokinetic analysis

Twenty-one New Zealand albino rabbits weighing 2.0–2.4 kg were used in this study. The rabbits were anesthetized with an ear intravenous injection of 10% chloral hydrate solution (3 mL/kg), while tropicamide ophthalmic solution was applied to dilate the rabbit pupils. Before intravitreal injection, oxybuprocaine hydrochloride eyedrops was used as a topical anesthetic for eyes. Subsequently, the left eyes were intravitreally injected with 50 μL Bev-MVLs (1.25 mg bevacizumab), whereas the right eyes were injected with Bev-S (1.25 mg bevacizumab) 2.5 mm posterior to the limbus at the superotemporal quadrant using a 30-gauge needle. Both eyes were monitored for possible inflammation. Three rabbits were sacrificed through an overdose of intravenous chloral hydrate at days 3, 7, 14, 21, 28, 42, and 56, respectively. The vitreous and aqueous humors were withdrawn using 1 mL syringe. All the samples were tested by ELISA.

### Laser induction and evaluation of CNV in rats

Male Brown-Norway (BN) rats weighing 200–230 g were used in this study. All the rat eyes were examined for the normal anterior segment and eyebase before experiments. The rats were anesthetized by intraperitoneally injecting 10% chloral hydrate solution (0.3 mL/100 g), while tropicamide ophthalmic solution was used to dilate rat pupils. Laser irradiation (532 nm; Vision One, Lumenis, Santa Clara, CA, USA) was delivered by a slit lamp (180 mW, 60 μm, 100 ms). Four to eight laser spots were applied in the right eyes with 2–3 disk diameters from the optic nerve head, while the left eyes served as control. The bubble formation indicated the induction of CNV through a rupture of Bruch’s membrane. The formation and development of CNV were monitored using optical coherence tomography (OCT; Spectralis OCT, Heidelberg Engineering, Heidelberg, Germany) and fluorescein fundus angiography (FFA; TRC-50IX, Topcon, Tokyo, Japan). OCT and FFA images were obtained at days 7, 14, and 21 after laser photocoagulation.

### Histologic examination

At 7 days after laser photocoagulation, BN rats were intravitreally injected with 0.25 mg/10 μL of bevacizumab. Five rats were treated with Bev-MVLs and other five were treated with Bev-S. All the animals were killed after 28 days of treatment. Posterior segment tissue samples of eyes were dehydrated and embedded in paraffin before cryostat sectioning into 4-μm thickness. Tissue sections were stained with hematoxylin and eosin (H&E) and examined using light microscopy.

### Data analysis

Statistical analysis was performed using Student’s *t*-test. For pharmacokinetic data analysis, the bevacizumab concentration-time data in both vitreous and aqueous humor were evaluated using non-compartmental methods. All the experimental data were expressed as mean ± SD. Statistically significant differences were set as *p* < .05 and highly significant differences were set as *p* < .01.

## Results

### Determination of the activity of bevacizumab

**Figure 1.**
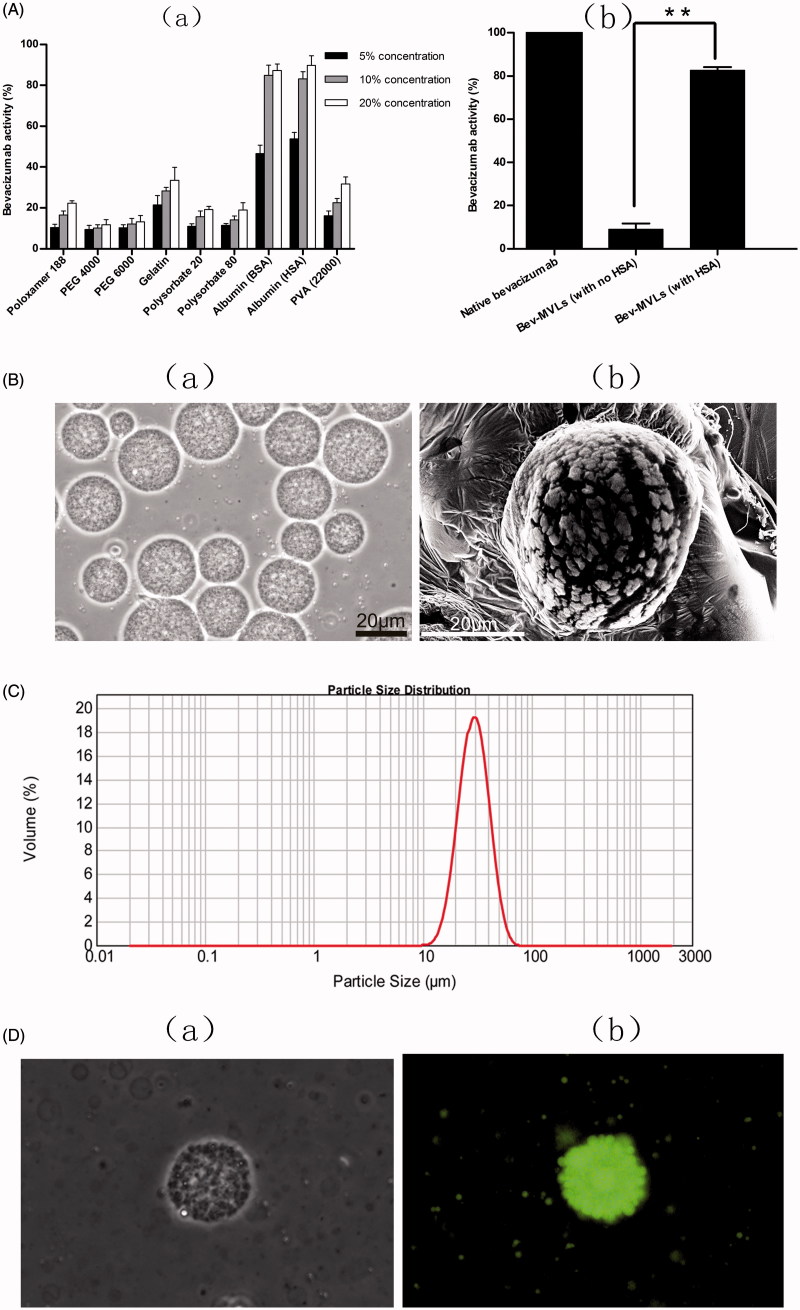
(A) The biological activity of bevacizumab: (a) the effect of various stabilizer on bevacizumab activity; (b) the bevacizumab activity of Bev-MVLs containing 10% HAS was statistically significantly different from without HAS (mean ± SD, *n* = 3, **p < .01). (B) The morphological examination of Bev-MVLs: (a) light micrograph of Bev-MVLs particles at 400× magnification; (b) SEM image of Bev-MVL particles. (C) Particle size distribution of Bev-MVLs. (D) The morphology of FB-MVLs was observed at 400× magnification (a) bright-field image; (b) fluorescent image.

In this study, several additives were evaluated in preserving the bioactivity of bevacizumab during emulsification. Figure 1(A)-a shows a reduction of bevacizumab activity during emulsification process. However, the activity of bevacizumab gradually increased toward the high concentrations of additive. The bevacizumab activities in all the additive groups were below 40%, except for BSA and HSA groups. These results indicated that the emulsification process adversely affected the antibody activity, especially in the presence of organic/aqueous interface. Hence, 10% BSA or HSA preserved the activity of bevacizumab up to 80%, PVA 22000 or 20% gelatin showed lower protective ability (up to 30% activity), followed by polyethylene glycol (PEG), polysorbate, and poloxamer. Meanwhile, Figure 1(A)-b demonstrates the antibody activity of Bev-MVLs with and without HSA. It is clearly shown that the antibody activity of Bev-MLVs with HSA was well preserved up to 80%, whereas Bev-MVLs without HSA exert only 9% bioactivity. These results suggest that albumin can effectively protect against denaturation and aggregation of antibodies. Since our ultimate goal is to prepare a working formulation for clinical usage, HSA was selected in this study.

### Preparation and characterization of Bev-MVLs

The morphology of Bev-MVLs observed by an optical microscope at 400× magnification is shown in Figure 1(B)-a. The particles of Bev-MVLs exhibited spherical morphology without good adherence and lipid debris, as well as uniform and smooth appearances. The SEM image and the size distribution profile of Bev-MVLs are shown in Figure 1(B)-b and 1(C), respectively. The findings revealed that Bev-MVLs had monomodal particle size distribution, median size of 29.5 μm, and 90% of the particles ranged in size from 11.9 to 43.6 μm. These results implied that the particles of Bev-MVLs had a regular shape and narrow size distribution.

### Fluorescence microscopy studies

FB was preliminary evaluated through protein concentration and F:P ratio (FITC:protein). Protein concentration was 2.28 ± 0.26 mg/mL, while F:P ratio was 3.58 ± 0.43. The results indicated that each antibody molecule attached 3 or 4 fluorescent molecules. FB is subjected to combination of lysine and thiocarbamide from FITC, which forms the conjugate of FITC-protein.

The morphology of FB-MVLs was observed to identify the entrapment of bevacizumab. Figure 1(D)-a and -b show the bright-field and fluorescent images for their particles. We found that MVLs displayed the aggregation of vesicles, and the multiple compartments formed particles with a roughly spherical shape. Fluorescent image showed that the spherical particles emitted green fluorescence, which indicated the entrapment potential of FB in MVLs.

### Encapsulation efficiency and *in vitro* release profiles

**Figure 2.**
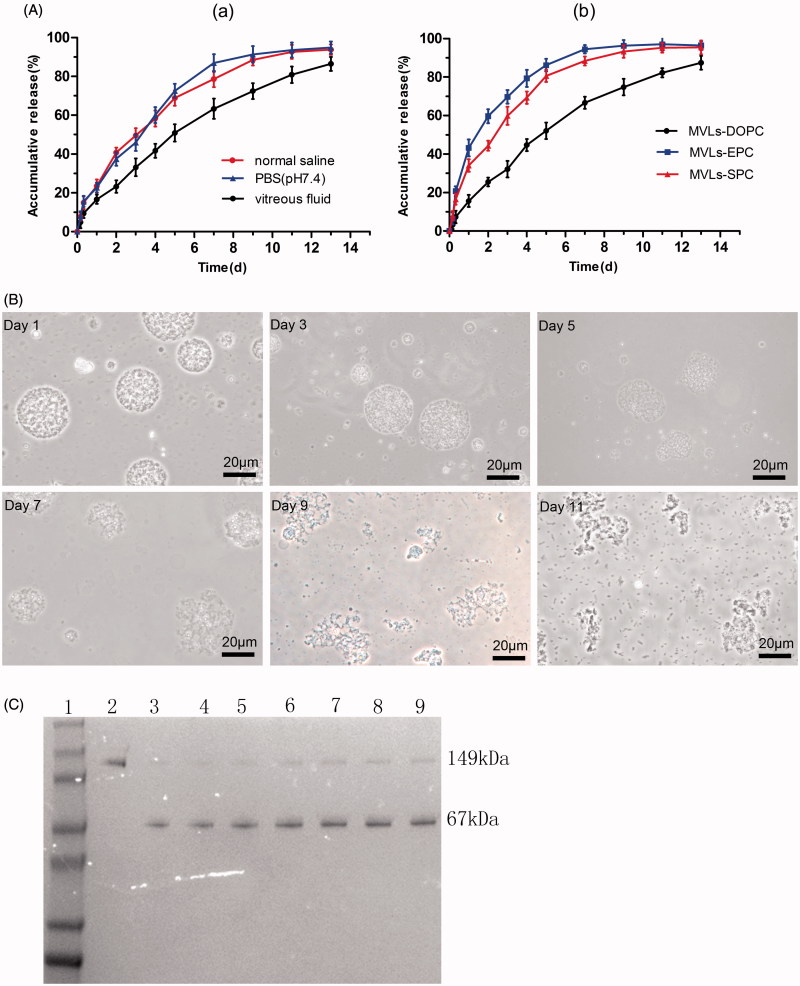
(A) *In vitro* release of Bev-MVLs (a) in three different mediums: normal saline, PBS (pH 7.4), and vitreous fluids; (b) coupled with different phospholipids (DOPC, EPC, and SPC) in vitreous fluids (mean ± SD, *n* = 3). (B) The morphological change of MVLs-DOPC at days 1, 3, 5, 7, 9, and 11 in vitreous fluids. (C) The results of SDS-PAGE for bevacizumab released from MVLs at scheduled intervals. Line 1: Marker; Line 2: free bevacizumab as a reference; Lines 3–9: bevacizumab released from MVLs at days 13, 11, 9, 7, 5, 3, and 1.

The encapsulation efficiency of Bev-MVLs was high (80.65 ± 4.42%), which may be attributed to the unique structure of particles. *In vitro* release profiles of Bev-MVLs in three different mediums are shown in Figure 2(A)-a. Approximately, 80% of bevacizumab was released in vitreous fluids (50% rabbit vitreous fluid +50% PBS) at day 13. Meanwhile, the initial burst releases of MVLs were lower than 30% in the three mediums. Figure 2(A)-b shows the release profiles of Bev-MVLs containing different phospholipids (e.g. DOPC, EPC, and SPC) in vitreous fluids. At the first day, the releases of MVLs were 19.7 ± 3.1%, 43.2 ± 4.4%, and 29.1 ± 3.2%, respectively. The release rate of MVLs-DOPC was significantly lower than those of MVLs-EPC and MVLs-SPC.

The model parameters for the release of MVLs are shown in Table 1. These results suggested that the first-order equation was the optimal equation of MVLs. As calculated from the Ritger–Peppas equations, the diffusion indexes (*n*) of MVLs-DOPC, MVLs-EPC, and MVLs-SPC were 0.741, 0.559, and 0.556, respectively. Notably, if 0.45 < *n* < 0.89, the mechanisms of drug release occurs mainly by diffusion and erosion, whereas *n* < 0.45 represents the Fick’s diffusion. As a result, the sustained release of Bev-MVLs acted primarily through diffusion and erosion mechanisms.

**Table 1.** The model parameters for the release of MVLs.

	Model	Equation	*R*^2^	*n*
MVLs-DOPC	Zero order	Q = 6.917*t* + 9.008	0.947	
	First order	Q = 105.729 (1-e^−0.137^*^t^*)	0.997	
	Higuchi	Q = 26.517*t*^1/2^ − 7.474	0.985	
	Ritger–Peppas	lnQ =0.741lnt +2.696	0.992	0.741
	Weibull	lnln1/(1 − Q/100)) = 0.920lnt −1.741	0.989	
MVLs-EPC	Zero order	Q = 6.907*t* + 30.514	0.687	
	First order	Q = 96.036 (1-e^−0.492^*^t^*)	0.991	
	Higuchi	Q = 27.872*t*^1/2^ + 12.558	0.874	
	Ritger–Peppas	lnQ =0.559lnt +3.450	0.884	
	Weibull	lnln1/(1 − Q/100) = 0.868lnt −0.759	0.976	0.559
MVLs-SPC	Zero order	Q = 7.17*t* + 23.921	0.788	
	First order	Q = 96.963 (1-e^−0.340^*^t^*)	0.990	
	Higuchi	Q = 29.443*t*^1/2^ + 3.281	0.954	
	Ritger–Peppas	lnQ = 0.556lnt +3.355	0.961	0.556
	Weibull	lnln1/(1 − Q/100) = 0.835lnt −0.928	0.989	

Figure 2(B) shows the morphological changes of Bev-MVLs particles in vitreous fluids at days 1, 3, 5, 7, 9, and 11, respectively. The morphology of particles was smooth and round in shape, while the antibodies were released mainly through diffusion. The erosion of MVLs particles began to appear from days 3 to 5, in which the particles were swollen and out of shape. From days 7 to 11, particles were ruptured and bursted into a large amount of small fragments.

### Structural stability of bevacizumab released from MVLs

The structural stability of bevacizumab released from MVLs was investigated by using SDS-PAGE. As presented in Figure 2(C), the specific protein bands of bevacizumab and HSA were visualized at about 149 kDa and 67 kDa (lines 3–9). In addition, no bevacizumab aggregates or structural fractures were found. Therefore, the sustained release of bevacizumab at various time points was considered as stable.

### *In vivo* imaging in a rat model

**Figure 3.**
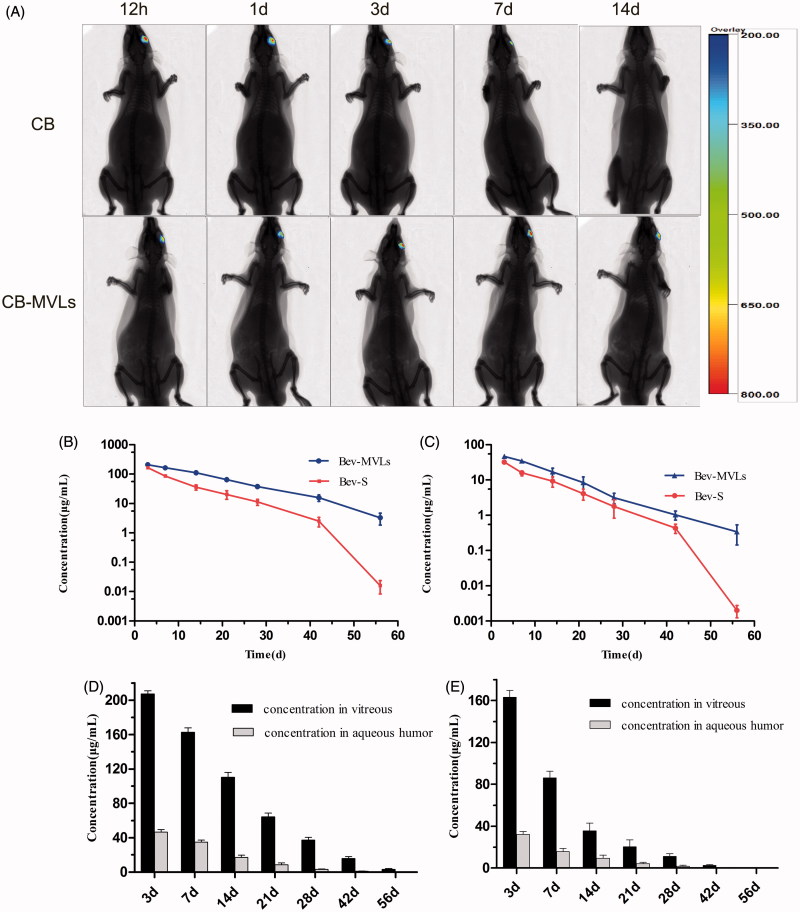
(A) *In vivo* imaging of SD rats after intravitreal injection of CB and CB-MVLs at 0.5, 1, 3, 7, and 14 days, respectively. (B) The concentrations of Bev-MVLs and Bev-S at 3, 7, 14, 21, 28, 42, and 56 days in vitreous humor, and (C) in aqueous humor. (D) The comparison of bevacizumab concentrations between the vitreous and aqueous humors after intravitreal injection of Bev-MVLs, and (E) of Bev-S (mean ± SD, *n* = 3).

*In vivo* imaging system has been applied to detect the targeting ability of drugs and to evaluate the time of drug release in experimental animals (Lee et al., 2011; Chen et al., 2014). Figure 3(A) represents the images of SD rats intravitreally injected with CB and CB-MVLs at 0.5, 1, 3, 7, and 14 days. In this series of images, we can easily observe the changes of fluorescence intensity in the left eyes. A weak fluorescence was observed at day 7, while no signal was detected at day 14 after injection with CB solution. In overall, CB solution group exhibited a rapid decline of fluorescence signals. Interestingly, the elimination rate of CB-MVLs group was much slower, and a fluorescence signal was still detected in rat eyes at day 14. It is obvious that CB-MVLs may exhibit sustained release properties.

### Pharmacokinetics of bevacizumab after intravitreal injection in a rabbit model

As illustrated in Figure 3(B), the concentrations of bevacizumab in vitreous humor were 207.28 ± 6.35 μg/mL (Bev-MVLs-treated) and 163 ± 6.54 μg/mL (Bev-S-treated) at day 3. As time passed by, its concentration declined to 37.22 ± 5.59 μg/mL and 11.23 ± 2.57 μg/mL, respectively, in both eyes at day 28. The final concentrations remained at 3.24 ± 1.41 μg/mL and 0.016 ± 0.01 μg/mL, respectively, at day 56. On the other hand, the concentrations of bevacizumab in aqueous humor reached 46.61 ± 5.16 and 32.22 ± 2.84 μg/mL at day 3 after intravitreal injection of Bev-MVLs and Bev-S, respectively. At day 28, its concentrations reduced to 3.16 ± 1.04 μg/mL and 1.78 ± 0.96 μg/mL, and the final concentrations were 0.34 ± 0.19 μg/mL and <0.01 μg/mL at day 56 (Figure 3(C)). The comparison of bevacizumab concentrations between the vitreous and aqueous humors after intravitreal injection with Bev-MVLs and Bev-S are presented in Figure 3(D,E), respectively.

In Table 2, the *t*_1/2_ values for Bev-MVLs and Bev-S were 8.84 ± 1.01 days and 4.45 ± 0.20 days in vitreous humor, and 8.26 ± 2.01 days and 4.23 ± 0.13 days in aqueous humor, respectively. These results further indicated that Bev-MVLs had a longer elimination half-life. Additionally, Bev-MVLs had much higher levels of bioavailability than those of Bev-S. The AUC_0–_*_t_* of Bev-MVLs was 1-fold greater than Bev-S, in the vitreous humor (3474.33 ± 255.95 vs. 1587.09 ± 184.26 μg/mL × days) and aqueous humor (582.38 ± 109.08 vs. 318.44 ± 60.21 μg/mL × days). According to mean residence time (MRT) values, Bev-MVLs could significantly improve the drug residence time in vitreous humor as compared to Bev-S (*p* < .05).

**Table 2.** Pharmacokinetic parameters of Bev-MVLs and Bev-S in the vitreous and aqueous humors following intravitreal injection (mean ± SD, *n* = 3).

	Pharmacokinetic parameters	Bev-MVLs	Bev-S
Vitreous humor	*t*_1/2_ (days)	8.84 ± 1.01*	4.45 ± 0.20
	*C*_max_ (μg/mL)	207.28 ± 6.35*	163.11 ± 6.54
	AUC_0–t_ (μg/mL × days)	3474.33 ± 255.95*	1587.09 ± 184.26
	MRT_0–_*_t_* (days)	15.03 ± 0.95*	10.19 ± 0.77
	*t*_1/2_ (days)	8.26 ± 2.0*	4.23 ± 0.13
Aqueous humor	*C*_max_ (μg/mL)	46.61 ± 5.16	32.22 ± 2.84
	AUC_0–_*_t_* (μg/mL × days)	582.38 ± 109.08*	318.44 ± 60.21
	MRT_0–_*_t_* (days)	11.33 ± 0.89	10.05 ± 0.95

*p < .05.

### Evaluation of CNV in rats

**Figure 4.**
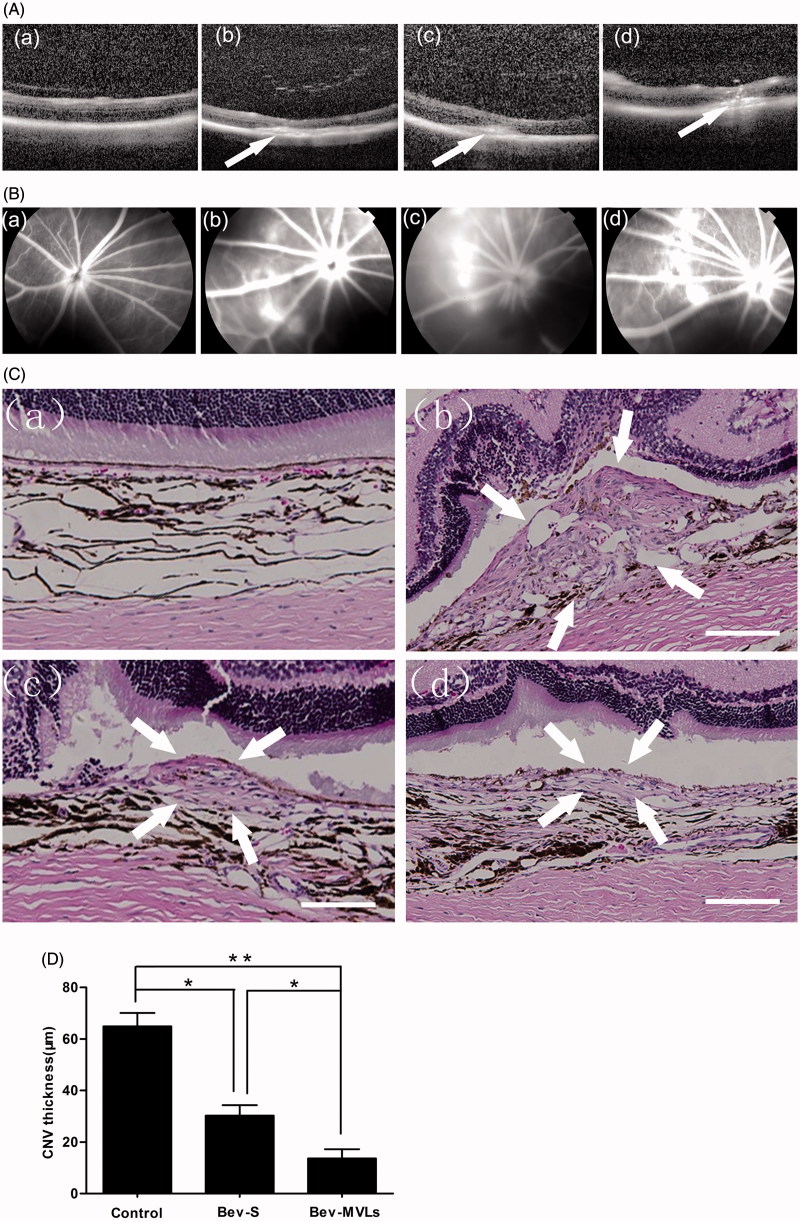
(A) OCT images of intraretinal layers cross-section (a) before photocoagulation; (b) 7 days after photocoagulation; (c) 14 days after photocoagulation; and (d) 21 days after photocoagulation. (B) FFA images: (a) before photocoagulation; (b) 7 days after photocoagulation; (c) 14 days after photocoagulation; and (d) 21 days after photocoagulation. (C) Histological images: (a) normal group without laser induction; (b) control group with 35 days after laser induction; (c) Bev-S group with 28 days after administration; and (d) Bev-MVLs group with 28 days after administration (white arrows indicate CNV lesion margins, scale bar, 50 μm). (D) BN rats treated with either Bev-S or Bev-MVLs had significantly thinner neovascular membranes than those in the control group (*p < .05, **p < .01, mean ± SD, *n* = 5).

The OCT images are shown in Figure 4(A). Intraretinal layers of normal BN rats were normal and recognizable, and CNV was gradually generated by the rupture of Bruch’s membrane at day 7 after photocoagulation. At days 14 and 21 after laser photocoagulation, the thickness of CNV membranes was continuously increased, which led to the spindle-shaped CNV lesion. FFA is another method for examining CNV formation. Figure 4(B) illustrates the changes of brightness in photocoagulation area in conjunction with the degree of fluorescein leakage. A mild fluorescein leakage appeared at day 7 after photocoagulation. Moreover, the intensities of fluorescein leakage were significant increased at days 14 and 21, and the photocoagulation area was presented as disc-shaped fluorescein.

Both of the OCT and FFA images confirmed that the thickness of CNV membrane was related to the brightness and area of fluorescein leakage within the retina. In particular, the brighter and larger the photocoagulation area, the thicker the CNV membrane. At day 14 after photocoagulation, CNV was initially formed and reached a peak at day 21.

### Histologic studies

As compared to the histological image of CNV before laser induction (Figure 4C-a), the neovascular membranes were generated and located in the subretinal space with numerous new vessels at 35 days after photocoagulation and without any treatment (Figure 4C-b). After 28 days of treatment with Bev-S and Bev-MVLs, the neovascular membranes were thinner and newly formed vessels were observed beneath the retina (Figure 4C-c, d). As shown in Figure 4(D), the mean diameters of the CNV lesion in Bev-S (30.3 ± 4.1 μm) and Bev-MVLs (13.6 ± 3.7 μm) treatment groups were smaller than those in a control group (64.9 ± 5.2 μm).

## Discussion

To the best of our knowledge, anti-angiogenesis therapy is commonly used for the treatment of neovascular AMD (Zampros et al., 2012). Anti-VEGF monoclonal antibody (bevacizumab) can inhibit the formation of new blood vessels, and greatly improve the patient’s vision. However, the activity of bevacizumab can be affected by hydrophobic surfaces, water-organic solvents, temperature, pH, etc. Therefore, in this study, the activity of bevacizumab was examined, due to the presence of organic/aqueous interface during the preparation of MVLs. Several pharmaceutical additives and protein stabilizers such as PEG, PVA, polysorbate, poloxamer, gelatin, and albumin have been extensively used to preserve the bioactivity of protein drugs during emulsification (Shimizu & Smith, 2004; Bilati et al., 2005; Son et al., 2009). However, the choice of such pharmaceutical additives is lacking, since not all stabilizer can be used to protect the bioactivity of protein drugs (Cleland & Jones, 1996; Meinel et al., 2001; Kang et al., 2002). In this study, albumin was considered an excellent protective agent to improve the stability of bevacizumab. As a stabilizer, albumin can substitute antibody in the organic/aqueous interface and prevent the absorption of bevacizumab onto the interface. This stabilizer not only exerts protective effects against denaturation and aggregation of bevacizumab, but also serves as an antibody carrier and affects the controlled release of bevacizumab.

Bev-MVLs are mainly constituted of synthetic amphipathic lipids, neutral oil, cholesterol, and stabilizers. Their internal structure is divided into multiple aqueous chambers, containing non-concentric lipid bilayers separated by 95% of water (Ye et al., 2000). To the best of our knowledge, negatively charged lipids can increase the interlamellar distance between successive bilayer of MVLs, and thus generate a high captured volume. The charged lipids also produce electrostatic repulsion, which are known to prevent aggregation (Kim et al., 1983). Neutral oil is the essential component of MVLs, enduring in the joints of each vesicle and eventually becomes part of the corners or edges to stabilize the membrane boundaries (And et al., 1996; Ellena et al., 1999). Interestingly, the amount of neutral oil used can modulate the duration of MVLs (Langston et al., 2003). Besides, cholesterol has been reported to retard the clearance and prolong the half-life of MVLs (Meisner & Mezei, 1995). Hence, MVLs are able to retain the water-soluble low-molecular-weight drug compounds and improve the encapsulation efficiency of water-soluble drugs. Despite their great potential as delivery systems for bioactive compounds (Abrishami et al., 2009), MVLs can overcome the low aqueous entrapment with less than 4 μL/μmol of lipid as compared to the conventional liposomes (Meyer et al., 1994). Furthermore, MVLs have the capability of protecting bevacizumab from physical and chemical inactivation under specific environmental conditions.

*In vitro* results demonstrated that the release rate of MVLs in vitreous fluids was lower as compared to NS and PBS (pH 7.4). More importantly, MVLs–DOPC formulations had a lower initial burst release and longer release time than natural lecithin. Generally, PBS has been used as release medium to maintain the stability of phospholipids (Jain et al., 2005; Zhang et al., 2016). In this study, we found that Bev-MVLs could prolong the release of drug in vitreous fluids for more than 13 days, suggesting that Bev-MVLs were more stable than PBS and NS. Such divergent results might be related to the consistency of release mediums. It has been reported that the saturation degree of phospholipids can determine the rigidity degree of liposomes (Holzschuh et al., 2018). Therefore, synthetic phospholipids are expected to have a greater tendency to prolong drug release than natural lecithin. As mentioned above, MVLs possess a unique structure of nonconcentric lipid chambers, which allows drugs to pass through their vesicles and diffuse into the release medium. Such features would definitely contribute to the prolonged release of bevacizumab. Furthermore, the observed morphological changes in MVLs particles could be explained by the processes of matrix erosion and vesicle rupture. The underlying mechanisms of MVLs may be different from other synthetic polymer-based nanoparticles and microspheres (Chen et al., 2014).

*In vivo* pharmacokinetics results indicated that Bev-MVLs could release bevacizumab for more than 56 days in vitreous and aqueous humors in a sustained release manner. A previous study showed that 0.5 μg/mL of bevacizumab is required to completely block VEGF induced endothelial cell proliferation (Wang et al., 2004). In this study, the bevacizumab concentration of Bev-MVLs in vitreous humor was 3.24 ± 1.41 μg/mL (>0.5 μg/mL) at day 56, and this concentration was higher than that of Bev-S. In fact, Bev-MVLs can maintain sustained drug release and effective therapeutic levels, lasting more than 56 days in rabbit vitreous humor. However, the results obtained in rabbit model may be inconsistent with those in human subjects (Nomoto et al., 2009). Therefore, the pharmacokinetic behavior of bevacizumab in rabbit eyes may be slightly differed from that in human eyes. The vitreous volume of human is approximately 3-fold larger than rabbit (4.5 mL vs. 1.5 mL). Hence, most of the drugs may need a larger volume of distribution, and a longer terminal half-life.

High energy laser photocoagulation technique is widely used to induce CNV through the disruption of Bruch’s membrane in animal models (Kamizuru et al., 2001). The pathophysiology of laser-induced CNV model may appear to be slightly different from the natural process of CNV disease, but the obtained pathological outcomes are always consistent. Both Bev-S and Bev-MVLs significantly inhibited the thickness of CNV lesions. Moreover, the thickness of CNV lesion in Bev-MVLs treatment group was significantly thin as compared to Bev-S treatment group (*p* < .05). It is implied that Bev-MVLs could extend the half-life of bevacizumab in vitreous humor of BN rats. In addition, Bev-MVLs exhibited a sustained release in vitreous humor, through a complete binding with the redundant VEGF. On the other hand, the half-life of Bev-S was relatively shorter. Bev-S concentration below the minimum blocking concentration of VEGF did not effectively inhibit the growth of neovascular membranes.

For patients with AMD, 1.25 mg of Avastin^®^ (bevacizumab) has been recommended to be administered by intravitreal injection every 4 weeks in the initial period of treatment. However, repeated bevacizumab injections and its associated complications still remain the major concerns. MVLs are excellent drug delivery systems with low toxicity, minimal immunogenicity, high biodegradability, and do not induce ocular irritation following an intravitreal injection. Thus, intravitreal injection of Bev-MVLs for the treatment of AMD can sustain and prolong the release of bevacizumab in vitreous, and possibly decrease the number of injections (e.g. once every 2 months or even longer). Consequently, the occurrence of side effects and complications from bevacizumab such as vitreous hemorrhage, endophthalmitis, and cataract would be reduced, and lead to improving patient compliance to medication.

## Conclusions

In this study, Bev-MVLs with high encapsulation efficiency (>80%) were formulated, exhibiting sustained drug release effects *in vitro* and *in vivo*. The bioactivity of bevacizumab in MVLs was well preserved by albumin stabilizer. Bev-MVLs exhibited lower initial burst release and prolonged drug release in vitreous fluids, maintaining structural stability of bevacizumab. Moreover, Bev-MVLs could sustain the therapeutic levels of bevacizumab in vitreous humor more effectively and longer lasting than Bev-S. More importantly, Bev-MVLs significantly inhibited the thickness of CNV lesions in rat eyes, and their therapeutic effects were greater than Bev-S at 28 days after treatment. In conclusion, Bev-MVLs are promising sustained release drug delivery systems for the treatment of CNV and other ocular neovascular diseases.
